# Glycoinositolphospholipids from Trypanosomatids Subvert Nitric Oxide Production in *Rhodnius prolixus* Salivary Glands

**DOI:** 10.1371/journal.pone.0047285

**Published:** 2012-10-15

**Authors:** Felipe Gazos-Lopes, Rafael Dias Mesquita, Lívia Silva-Cardoso, Raquel Senna, Alan Barbosa Silveira, Willy Jablonka, Cecília Oliveira Cudischevitch, Alan Brito Carneiro, Ednildo Alcantara Machado, Luize G. Lima, Robson Queiroz Monteiro, Roberto Henrique Nussenzveig, Evelize Folly, Alexandre Romeiro, Jorick Vanbeselaere, Lucia Mendonça-Previato, José Osvaldo Previato, Jesus G. Valenzuela, José Marcos Chaves Ribeiro, Georgia Correa Atella, Mário Alberto Cardoso Silva-Neto

**Affiliations:** 1 Instituto de Bioquímica Médica, Universidade Federal do Rio de Janeiro, Rio de Janeiro, Brazil; 2 Instituto Federal de Educação, Ciência e Tecnologia do Rio de Janeiro, Rio de Janeiro, Brazil; 3 Instituto de Biofísica Carlos Chagas Filho, Universidade Federal do Rio de Janeiro, Rio de Janeiro, Brazil; 4 Department of Leukemia, M.D. Anderson Cancer Center, Houston, Texas, United States of America; 5 Universidade Federal Fluminense, Instituto de Biologia. Campus Valonguinho, Prédio do Instituto de Biologia, Departamento de Biologia Celular e Molecular, Centro, Niterói, Rio de Janeiro, Brasil; 6 Université de Lille 1, Unité de Glycobiologie Structurale et Fonctionnelle, Villeneuve d’Ascq, France; 7 Vector Molecular Biology Section, Laboratory of Malaria and Vector Research, National Institute of Allergy and Infectious Diseases, National Institutes of Health, Rockville, Maryland, United States of America; 8 Instituto Nacional de Ciência e Tecnologia em Entomologia Molecular (INCT-EM), Rio de Janeiro, Brazil; Federal University of São Paulo, Brazil

## Abstract

**Background:**

*Rhodnius prolixus* is a blood-sucking bug vector of *Trypanosoma cruzi* and *T*. *rangeli. T. cruzi* is transmitted by vector feces deposited close to the wound produced by insect mouthparts, whereas *T. rangeli* invades salivary glands and is inoculated into the host skin. Bug saliva contains a set of nitric oxide-binding proteins, called nitrophorins, which deliver NO to host vessels and ensure vasodilation and blood feeding. NO is generated by nitric oxide synthases (NOS) present in the epithelium of bug salivary glands. Thus, *T. rangeli* is in close contact with NO while in the salivary glands.

**Methodology/Principal Findings:**

Here we show by immunohistochemical, biochemical and molecular techniques that inositolphosphate-containing glycolipids from trypanosomatids downregulate NO synthesis in the salivary glands of *R. prolixus*. Injecting insects with *T. rangeli*-derived glycoinositolphospholipids (Tr GIPL) or *T. cruzi*-derived glycoinositolphospholipids (Tc GIPL) specifically decreased NO production. Salivary gland treatment with Tc GIPL blocks NO production without greatly affecting NOS mRNA levels. NOS protein is virtually absent from either Tr GIPL- or Tc GIPL-treated salivary glands. Evaluation of NO synthesis by using a fluorescent NO probe showed that *T. rangeli*-infected or Tc GIPL-treated glands do not show extensive labeling. The same effect is readily obtained by treatment of salivary glands with the classical protein tyrosine phosphatase (PTP) inhibitor, sodium orthovanadate (SO). This suggests that parasite GIPLs induce the inhibition of a salivary gland PTP. GIPLs specifically suppressed NO production and did not affect other anti-hemostatic properties of saliva, such as the anti-clotting and anti-platelet activities.

**Conclusions/Significance:**

Taken together, these data suggest that trypanosomatids have overcome NO generation using their surface GIPLs. Therefore, these molecules ensure parasite survival and may ultimately enhance parasite transmission.

## Introduction

Invasion of vector salivary glands is a mandatory step in the life cycle of several pathogens. This event ensures pathogen transmission to a vertebrate host in the next feeding cycle. However, by infecting the salivary glands, pathogens come into direct contact with vector saliva and with the anti-hemostatic factors it contains [1]. Thus, their survival in this new environment relies on their ability to avoid the action of potentially harmful anti-hemostatic factors. This may be achieved by the manipulation of the production of some of these factors by parasite-derived molecules. These processes will enhance the transfer of saliva (and parasites) to the vertebrate host skin. In fact, we can state that the outcome of vertebrate host infection is pre-determined by pathogen interactions that occur while they are still within the invertebrate host saliva. Nitric oxide synthase (NOS) is an enzyme that catalyzes the generation of nitric oxide (NO), NADP and L-citrulline, using L-arginine as substrate, NADPH as a cofactor and O_2_ as oxygen donor [2]. There are three known isoforms of this enzyme in vertebrates: a neuronal (nNOS or type 1), an inducible (iNOS or type 2) and an endothelial (eNOS or type 3) [3]. The type 1 and 3 isoforms are collectively known as constitutive NOS. NOS activity in an invertebrate animal was first described for the salivary glands of *Rhodnius prolixus,* a triatominae bug that is a vector of *Trypanosoma cruzi,* the etiological agent of Chagaś disease [4], and in the brain of *Schistocerca gregaria* [5]. *R. prolixus* is also the vector for *T. rangeli*, another trypanosomatid that is considered to be harmless to humans. *T. rangeli* is transmitted by bug saliva and is in close contact with an NO source until its transmission [6].

The role of NO in invertebrate immune response has been observed in several models [3], [7]. Early evidence that insects generate this molecule when infected with protozoan parasites was obtained in Anophelinae mosquitoes infected with *Plasmodium berghei* [8]. The NOS of the midgut of *S*. *stephensi* was monitored by semi-quantitative PCR at different days after infection. There was a large increase in gene expression in infected animals [8]. When the generation of NO was blocked using a NOS inhibitor, L-NAME, there was an increase in the number of parasites [8]. The ability of NO to eliminate parasites was also observed in gastropod hemocytes resistant to *Schistosoma mansoni* [9]. Harmful effects of NO in biological systems include the oxidation of heme groups and nitrosylation of some amino-acid residues, causing unscheduled changes in the secondary structure of proteins, and thus blocking parasite viability [10]. Finally, NO also acts as a signaling molecule, having a key role in the insect immune system [11].

Infection with *T. rangeli* inhibited the generation of NO in the salivary glands of *R. prolixus* [12]. However, the techniques used by those authors did not evaluate NOS activity, and its expression and inhibitory mechanisms were not described. It was later demonstrated that the administration of NOS inhibitors leads to an increase in *T. rangeli* parasitemia [12]. When NOS expression and NO generation were measured in different tissues of *R. prolixus* infected either with *T. rangeli, T. cruzi* or with lipopolysaccharide (LPS) [13], [14], each tissue responded differently. In general, LPS and *T. cruzi* infection increased the expression of NOS and no changes were detected with *T. rangeli* infection. However, salivary glands were not evaluated in that study. Since salivary glands are ultimately involved with parasite transmission and are the last tissue to be infected, a specific study of the effects of *T. rangeli* infection is still needed.

Besides its activities in the insect immune system, NO also facilitates the feeding of *R. prolixus*. This occurs when NO binds to the heme of the host soluble guanylate cyclase enzyme, which converts magnesium guanosine 5′-triphosphate into cGMP. The elevation of intracellular cGMP levels causes the smooth-muscle layer surrounding the vascular endothelium to relax, increasing the caliber of blood vessels. NO is also an inhibitor of platelet aggregation [15], [16]. Since the description of the presence of NO in the salivary glands of *R. prolixus*, it has been suggested that a group of proteins present in the saliva could bind to NO [15], [17]. Such proteins were found to reversibly bind NO, being named nitrophorins (NPs) [15], [18]. So far seven different NPs have been identified in *R. prolixus*, and are responsible for NO delivery to the vertebrate host bloodstream [19]. Therefore, the interaction between triatominae and trypanosomatids provides a unique host versus parasite model system where salivary NO plays a dual role, as both an anti-hemostatic molecule and as an immune-activated molecule.

Glycosylphosphatidylinositol (GPI)-anchored glycoproteins from the surface of trypanosomatids are key mediators of host-parasite interactions. This idea was first established through studies in *T. brucei* [20]. The surface of this parasite is covered by a dense layer of glycoproteins, and these molecules display high antigenic variation. These data were correlated with early work describing the ability of this parasite to avoid the immune response of their vertebrate hosts. Since then, many studies have described the effects of different glycoconjugates from trypanosomatids on the immune system of vertebrates, with emphasis on mammals [21], [22], [23], [24]. A common feature to almost all of these glycoconjugates is their attachment to the outer membrane of parasites through a GPI-anchor. The GPI-anchor consists of an inositolphospholipid skeleton bound to a conserved carbohydrate chain (Manα1-2Manα1-6Manα1-4GlcNα1-6myoinositol1-PO_4_-lipid). The lipid portion remains immersed in the cell membrane and can consist of either a glycerolipid or a ceramide [21]. Proteins may be attached to such anchors through a peptide bound between the protein’s C-terminus and either an ethanolaminephosphate or aminoethylphosphonate substituent branching out of the third mannose of the aforementioned conserved glycan chain.

GPI-anchors that are not linked to proteins or polysaccharides are known as glycoinositolphospholipids (GIPLs). These molecules are found in large quantities on the surface of trypanosomatids and, in general, there may be more than 10^7^ GIPL copies per cell in *T. cruzi* [21]. They can also be found on the surface of other parasites, such as *Plasmodium* [22], [23]. Studies have shown that parasite infection relies on bioactive GIPLs or GPI-anchors that modulate the mammalian immune system. These effects include the modulation of NO synthesis in macrophages, regulation of the levels of cytokines and cell-adhesion molecules and the manipulation of cell-signaling pathways, among many others [21]–[24]. Very little is known about what effects parasite GIPLs have on the cell signaling of insect vectors. Thus, it is clear that more studies on the effects of surface molecules from parasites on insect vectors are needed. The use of purified molecules allows experiments to be done in the absence of living parasites and provides new information on the relationship between parasites and vectors. Here, we provide for the first time evidence for the role of surface inositolphosphate-containing glycolipids (GIPLs) as molecules responsible for subverting NO synthesis in a blood-sucking arthropod.

## Results

### *T. rangeli* Blocks NO Production in Salivary Glands through a Mechanism that Involves its Surface GIPLs

**Figure 1:**
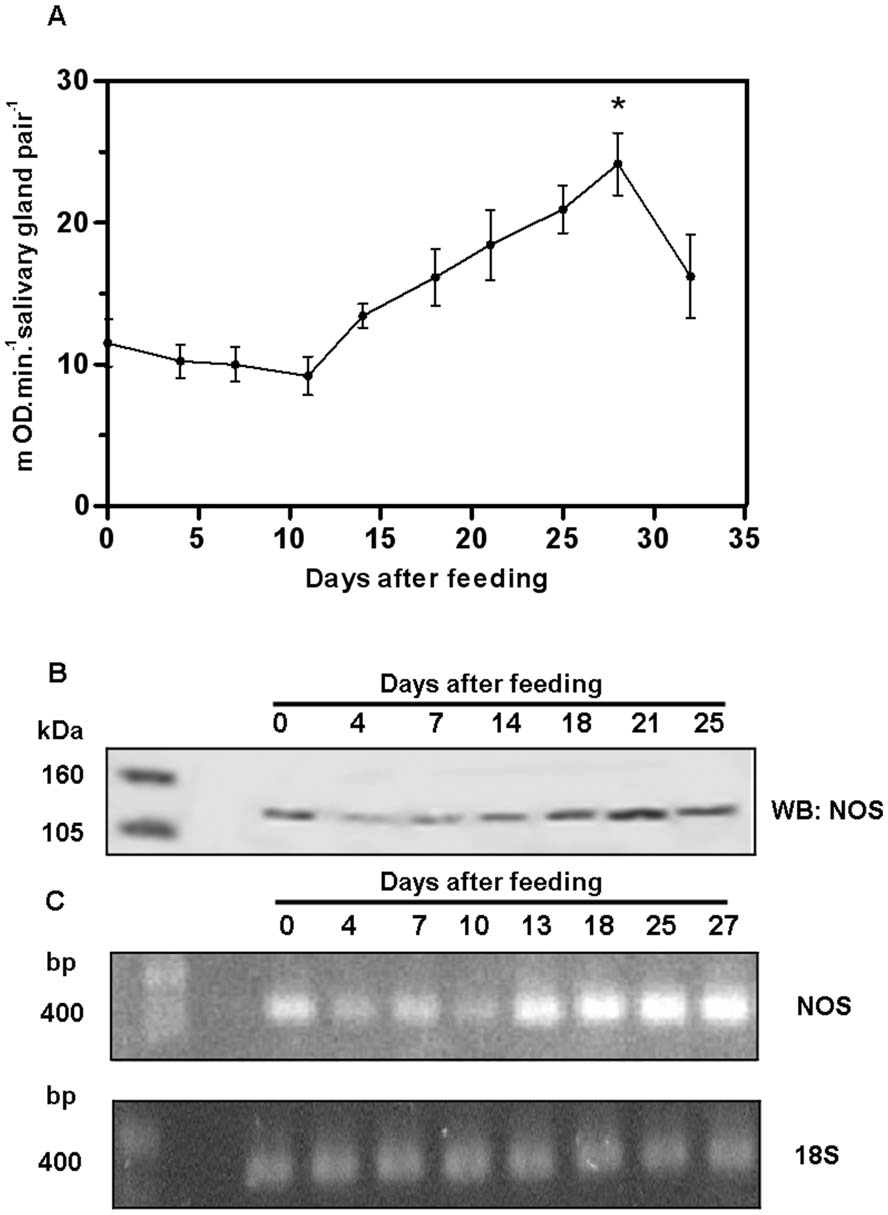
NADPH-diaphorase activity of NOS in *Rhodnius prolixus* salivary glands after a blood meal and the expression of NOS. A. Salivary glands were dissected in different days after blood feeding and evaluated for NOS NAPDH-diaphorase activity. Salivary glands were assayed in 10 mM Tris-HCl pH 8,0, 0,05 M NaCl, 0,1%, Triton X-100, 1 mM CaCl_2_, 5 µM FAD, 1 mM NADPH and 0,5 mg/mL MTT. MTT reduction was followed at 540 nm for 30 min at 37°C. Also samples were obtained and NOS content evaluated by Western blotting. Each point is the average and SE of 05 different experiments. B. Immunoblotting using an anti-NOS antibody. Blottings were developed with the use of a secondary antibody conjugated to alkaline phosphatase in the presence of the substrate Western Blue. Molecular mass markers are indicated at the left. C. Upper panel**,** total RNA from the salivary glands at different days after feeding was isolated and cDNA was synthesized. Samples were then analyzed by semi-quantitative PCR with temperatures of 55, 72 and 94°C for 27 cycles with primers for NOS. Lower panel, analysis of 18 S RNA levels. In this case reaction occurred for 25 cycles. The products of reactions shown on panels C were separated on agarose gel 1.4% stained with ethidium bromide and photographed under ultraviolet light. Molecular mass standards are indicated at the left.

**Figure 2:**
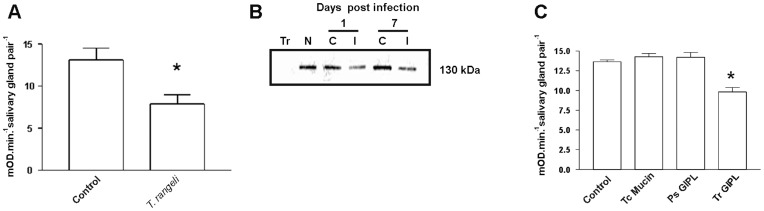
Infection with *T. rangeli* reduces the NOS activity and the levels of NOS protein in the salivary glands of *R. prolixus*. A. *Rhodnius* were dissected 7 days after control injection of water or *T. rangeli* and assayed for NADPH-diaphorase activity. Results from three experiments were evaluated statistically using the Student t test (* p<0.05). B. Salivary gland extracts from control or *T. rangeli-*injected insects were fractionated by SDS-PAGE and transferred to a nitrocellulose membrane. The membranes were incubated with primary antibody anti-NOS and then with an anti-rabbit antibody conjugated to alkaline phosphatase. This experiment was performed three times. Tr, *Trypanosoma rangeli* cells evalutated for NOS blotting. N, salivary glands from non-injected insects. C, control salivary glands from insects injected with water. I, Salivary glands from *T. rangeli*-injected insects. C. NADPH-diaphorase activity was measured in salivary gland extracts of salivary glands three days after injection with 100 ng of glycolipids from either *T. rangeli* (Tr GIPL), *P. serpens (*Ps GIPL*)* or *T. cruzi* eGPI-mucin (Tc Mucin). The experiment was performed three times and analyzed by ANOVA (* p<0.05).

**Figure 3:**
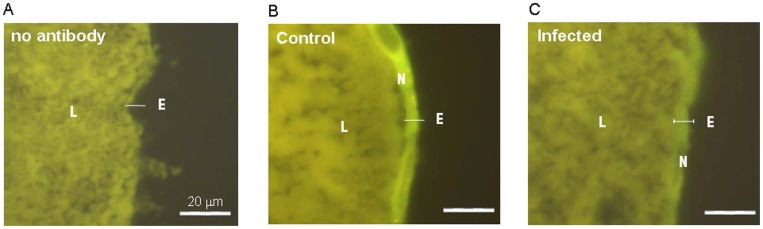
*T. rangeli* infection downregulates NOS production. *Rhodnius* were infected with *T. rangeli* and three days later salivary glands were dissected and analyzed by immunocytochemistry using anti-NOS. A. no antibodies. B. Control salivary glands developed with anti-NOS and a secondary antibody. C. Infected salivary glands developed with both antibodies. (E), salivary gland epithelia, (L), salivary gland lumen, (N), nucleus of salivary gland epithelial cells.

**Figure 4:**
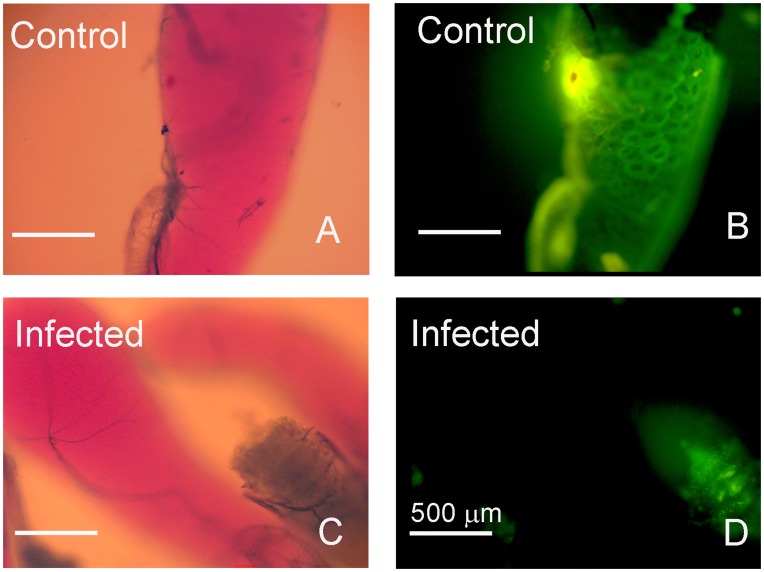
*T. rangeli* infection downregulates NOS synthesis. *Rhodnius* were infected with *T. rangeli* and three days later salivary glands were dissected and incubated in the presence of the NO fluorescent probe DAF-FM. A, C are contrast-phase imaging of B and D, respectively. A. Control salivary gland. B. DAF-FM fluorescent image of a control salivary gland shown on A. C. Infected salivary gland. D. DAF-FM fluorescence image of an infected salivary gland shown on C.

Following a blood meal, *Rhodnius* ´ salivary glands are continuously refilled by the secretion of anti-hemostatic molecules synthesized from salivary-gland epithelia. In *R. prolixus* the nitric oxide-binding proteins, nitrophorins (NP), are synthesized in the first part of the feeding cycle in the 4^th^ instar, and later on after moulting are readily loaded with NO [25]. NO synthase (NOS) activity in *Rhodnius* salivary glands can be reliably evaluated by NADPH-diaphorase activity [25]. The surge on NADPH-diaphorase activity as a function of NOS synthase activity was due to an increase in the expression of the NO synthase gene itself, as evaluated by enzyme activity and immunoblotting against this enzyme (Figures 1A and 1B). Semi-quantitative PCR experiments suggested that the increase in NOS expression occurred following an increase in the levels of mRNA coding for this enzyme (Figure 1C). In order to verify the role of parasite infection in this NO-NOS model, in the next experiments we have separately evaluated the levels of NOS and NO synthesis in isolated salivary glands obtained from both control and *T. rangeli*-infected insects as shown on Figure 2. Figures 2A and 2B show a decrease in NADPH-diaphorase activity and NOS protein in infected salivary glands, as evaluated by western blotting and enzyme assay data. To test for the role of parasite-derived surface molecules in the suppression of NADPH-diaphorase activity we have next evaluated the effect of *T*. *cruzi*-derived GIPLs, a GPI-anchored mucin-like glycoprotein isolated from epimastigote forms (eGPI-mucin) from *T*. *cruzi* surface glycoconjugate, GIPLs isolated from *T. rangeli* surface (Tr GIPL) and a GIPL preparation isolated from *Phytomonas serpens*, a trypanosomatid that infects plant [26], [27], [28]. Figure 2C shows that only (Tr GIPL) suppressed NADPH-diaphorase activity. Tr GIPL chemical composition was then determined (Table 1). Figure 3 shows that the levels of NOS are decreased in infected salivary glands specifically in the neighboring epithelial cells around the gland lumen, as evaluated through immunocytochemistry (Figure 3). This confirmed that *T. rangeli*-mediated suppression of NADPH-diaphorase activity (Figure 1A) and NOS expression (Figure 2B) specifically occurred on salivary gland epithelium, which is the major site of NOS localization in this tissue. So far, NO synthesis was indirectly evaluated by the NADPH-diaphorase assay, and a direct evaluation assay of NO production was not conducted. Thus, isolated salivary glands from control and infected insects were loaded with the NO-sensor DAF-FM and evaluated for tissue integrity (Figures 4A and 4C). *T. rangeli* infection leads to a large decrease in NO production (Figures 4B and 4D).

**Table 1:** Chemical composition of GIPL purified from *T*. *rangeli* (Tr GIPL).

Component	Molar Ratio
**Monosaccharide** a	
Mannose	4.08
Galactose	1.00
Glucosamine	1.04
*Myo*-Inositol	1.00
**Lipid**	
Fatty acid^b^	
C16∶0	3.00
C18∶0	2.00
C24∶0	1.00
Long Chain Base^c^	
Sphingosine	1.04
Sphinganine	1.00

a Determined by GC as trimethylsilyl derivatives of methylglycosides.

b Determined by GC and GC-MS as fatty acid methyl esters (FAMEs).

c Determined by GC and GC-MS after N-acetylation and trimethylsilylation.

**Figure 5:**
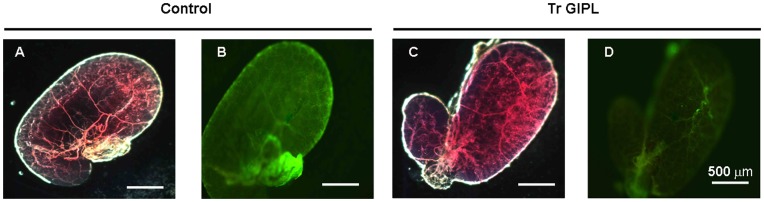
Tr GIPLs suppress NO synthesis. *Rhodnius* were injected either with 100 ng of Tr GIPL or water and three days later salivary glands were dissected and incubated with DAF-FM. A. Image of control salivary glands. B. DAF-FM fluorescent image of a control salivary gland. C. Image of salivary glands isolated from insects injected with Tr GIPL. D. DAF-FM fluorescence image of salivary glands isolated from insects injected with Tr GIPL.

**Figure 6:**
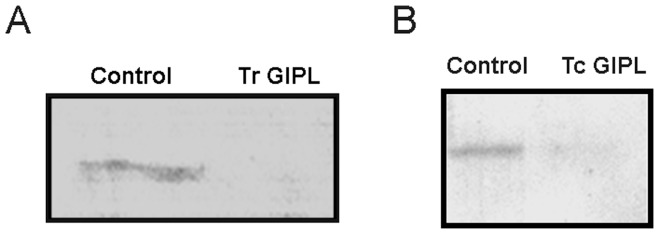
Glycolipids from *T. rangeli* and *T. cruzi* suppress NOS expression. *Rhodnius* were injected with either Tr GIPL or Tc GIPL and three days later salivary glands were dissected, homogenized and NOS expression evaluated by Western blotting. A. Western blotting against NOS in salivary glands obtained from control and insects injected with Tr GIPLs. B. Western blotting against NOS in salivary glands obtained from control and insects injected with Tc GIPLs.

In most pathogen x host cell interaction models parasite interaction with host cells and tissues occurs before invasion. Surface molecules are usually involved in such interaction and mediate the first signaling events leading to the suppression of host-mounted defenses against pathogens. As shown in the previous experiments, Tr GIPLs were able to decrease NADPH-diaphorase activity from salivary glands (Figure 2C). We next tested whether Tr GIPL could affect NO synthesis. Figure 5 shows that NO synthesis was suppressed in glands injected with Tr GIPL. Also, such effect is associated with the suppression of the level of NOS protein, since this protein is markedly reduced by treatment of salivary glands with Tr GIPL (Figure 6A). This effect was also seen in glands injected with *T. cruzi*-derived GIPLs (Tc GIPL) (Figure 6B). The amount of Tr GIPL obtained in our experiments was very low. Besides that, the same effects on NO biology in *Rhodnius* observed with Tr GIPL were also observed with Tc GIPL. Therefore, in the following experiments Tc-GIPL was used in order to detail GIPL effects on NO production in *Rhodnius*.

### Tc GIPL-induced Manipulation of NO Synthesis is Specific Towards this Anti-hemostatic Molecule and is Mediated by the Suppression of a Protein Tyrosine Phosphatase Activity

**Figure 7:**
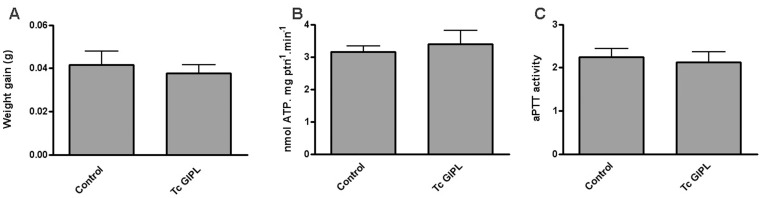
Tc GIPL does not affect regular blood feeding, anti-clotting and apyrase activity. *Rhodnius* injected or not with Tc GIPLs were evaluated for their ability to feed on blood. Parallel controls in each panel were obtained in insects inject with GIPL solvent. Three days after the injection insects were either allowed to feed on a rabbit ear or their salivary glands were dissected and evaluated for anti-hemostatic activities. A. Weight gain after blood feeding. B. Apyrase activity. C. aPTT activity. Data is the mean ± S.E. of three different experiments.

**Figure 8:**
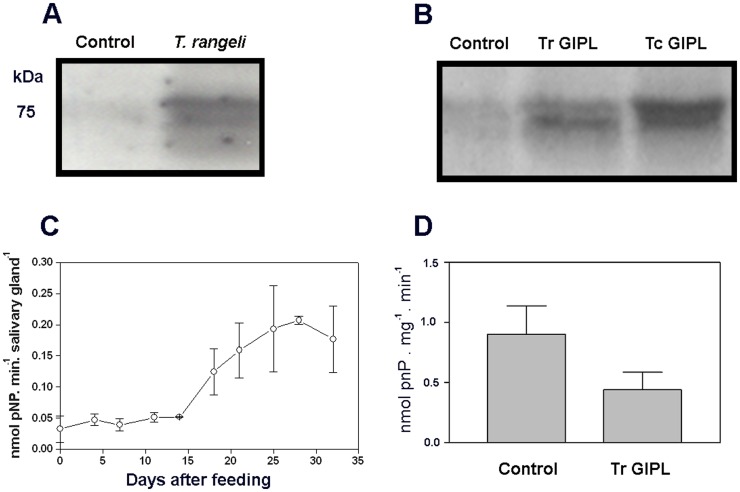
Glycolipid-mediated suppression of NO synthesis occurs through the manipulation of intracellular phosphorylation-dephosphorylation circuits. Intracellular circuits of protein-phosphorylation and dephosphorylation were evaluated through different assays. A. Salivary glands obtained from either control or *T. rangeli*-infected insects were dissected three days after the injection, homogeneized and phosphorylated in the presence of ^32^P-ATP followed by SDS-gel electrophoresis and autoradiograph. B. A similar experiment was conducted with salivary glands isolated from insects injected with Tr GIPL or Tc GIPL. C. Following a blood meal on rabbit ear salivary glands were dissected at different points in time. Total protein phosphatase activity was followed during the refilling cycle of salivary glands using pNPP as substrate. Data is the mean ± S.E. of three different experiments. D. Insects were injected with Tr GIPL and evaluated for protein phosphatase activity in the presence and in the absence of SO. The fraction of enzyme activity inhibited by SO in control and Tr GIPL-injected insects is shown. Data is the mean ± S.E. of three different experiments.

The following experiments were designed to obtain more detailed information regarding the effect of Tc GIPL on *Rhodnius* salivary glands. We first evaluated the effect of Tc GIPL of the production of overall anti-hemostatic molecules of *Rhodnius*, and in the ability of the insects to feed on blood. Figure 7A shows that Tc GIPL did not affect insect regular blood-feeding following days after treatment. Also, there was no change in total apyrase activity or clotting time for saliva from Tc GIPL-treated glands (Figures 7B and 7C). This indicates that the parasités manipulation of salivary gland anti-hemostatic components occurred by a specific intracellular signaling pathway that only disrupted NO biological circuits. Protein phosphorylation-dephosphorylation is a major mechanism of regulation of NO production in several models [2]. In order to obtain evidence that components of this signaling pathway are being manipulated, the following experiments were conducted. Figure 8A shows that *in-vitro* exposure of *T. rangeli*-infected glands extracts to ^32^P-ATP induced the phosphorylation of a major set of ∼75-kDa bands. Also, glands of insects treated with Tr GIPL or Tc GIPL showed a similar phosphorylated set of bands (Figure 8B). This result suggested that the phosphorylation circuits in both infected and glycolipid-treated glands were very similar. We next evaluated the role of protein dephosphorylation in GIPL-mediated NO suppression in salivary glands. We first tested whether the activity of protein tyrosine phosphatases (PTPs) are involved in the regulation of NO production [29]. Figure 8C shows that total PTP activity increased during the refilling cycle, being the highest at 30 days, the time for maximal loading with NO (as demonstrated in Figure 1 for NOS). Salivary gland PTP activity is highly inhibited by sodium orthovanadate (SO), which is a classical inhibitor of PTPs [29], [30], [31], [32], [33]. Furthermore, injection of the insects with Tc GIPL decreased PTP activity in glands (Figure 8D).

**Figure 9:**
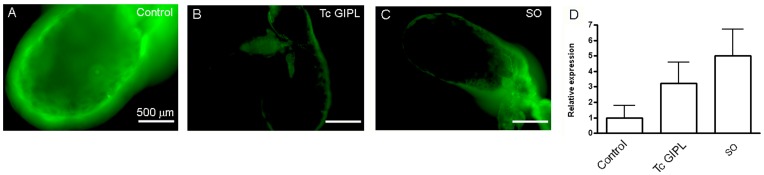
Tc GIPL-mediated suppression of NO synthesis is mediated through the inhibition of a protein tyrosine phosphatase. *Rhodnius* were injected with either water, Tc GIPLs or sodium orthovanadate (SO) and three days later analyzed for NO synthesis by DAF-FM fluorescence. Also samples were collected for the evaluation of NOS mRNA levels by RT-PCR. A. DAF-FM fluorescence image of control insects. B. DAF-FM fluorescence image form salivary glands dissected from GIPL-injected insects. C. DAF-FM fluorescence image from salivary glands dissected from SO-injected insects. D. RT-PCR analysis of NOS mRNA from control, Tc GIPL- and SO-injected insects. Data is the mean ± S.E. of three different experiments. The experiment was performed three times and analyzed by NOVA (p>0.05) which indicated that there is no statistically significant difference among groups.

Next, we evaluated whether SO could induce a decrease in NO synthesis as compared to control glands (Figure 9). Figure 9B and 9C show that Tc GIPL and SO treatment each reduced NO production to a similar extent. However, both interventions (Tc GIPL and SO) did not statistically affect the levels of mRNA for NOS (Figure 9D). Thus, it is likely that upon invasion of the salivary gland, parasite surface GIPLs inhibited a vector PTP that was essential to keep NOS mRNA continuously under translation and ultimately led to inhibition of NO synthesis.

## Discussion

The first published study addressing the role of glycolipids in NO biology during vector × parasite interaction used the *P. falciparum* × *An. stephensi* model [34]. In that study, it was demonstrated that GIPL concentrations between 0.25 and 2.5 µM were able to induce NOS expression at the same intensity as infection with live parasites. Nogueira et al. (2007), working in another model showed that administration of 100 nM of Tc GIPL inhibited the adhesion of these microorganisms to the digestive tract of bugs by 95% [35]. Possibly, parasite glycoconjugates interact with vector gut through lectins present in the peritrophic matrix found in most insects and in *Rhodnius* perimicrovilllar membrane [34], [36]. Another work also observed an interaction between *T. rangeli* and the salivary glands of *R. prolixus,* showing that *T. rangeli* incubation with certain types of carbohydrates inhibited parasite-salivary glands interaction. Incubation with salivary glands glycoconjugates also resulted in the same effect [37]. Thus, it may suggest that lectins and other receptors on the surface of *T. rangeli* and salivary glands of *R. prolixus* may be involved in the interaction between these two organisms.

In this study, the sugar, fatty acid and long-chain base compositions of GIPLs purified from *T. rangeli* have been elucidated (Table 1). It is known that trypanosomatids are phylogenetically close groups, and tend to have overall similar surface molecules. The species closest to *T. rangeli* that has had its surface glycoconjugates characterized is *T. cruzi* [21], [38]. Indeed, the Tr GIPL is a glycoinositolphosphosphingolipid whose composition is similar to Tc GIPL [39]. The comparison of the effects caused by administration of GIPLs from *T. cruzi* or *T. rangeli* on invertebrate host might bring new information on the biology of these two species. Since both species coexist in the same host, it is possible that in some stages of their life cycles, they may use similar strategies to suppress vector immune responses. Also, the interference of parasite species on one another resulting from the co-existence on the same host must be evaluated.

Our experiments suggested that the effects of live parasites or their glycolipids were due to the down regulation in NOS expression. Tr GIPL largely affected NOS protein expression and NO synthesis (Figures 2, 5). Furthermore, one may consider that there may be more than one isoform of NOS in the salivary glands of *R. prolixus*. In this situation, one NOS isoform might be constitutively expressed throughout the period of saliva replacement, while a second NOS isoform might be expressed only after insect molting. In this case, NP expression would be higher in the first days after blood feeding and dramatically lower following insect molting [18], [25]. Thus, there may be specific mechanisms that adjust NP synthesis to NO availability. The information regarding the presence of multiple NOS isoforms in *R. prolixus* is not available. However, if this is the case, potential effects of *T. rangeli* GIPLs on each isoform, resulting in partial inhibition of total levels of NOS in this model must be evaluated. In this case, glycolipids could inhibit the isoform with induced expression after molting, causing the total levels of NOS in the salivary glands to return to basal the levels found up to day 15. It is remarkable that the induction of NOS expression occurs after molting in the same phase where the PTP activity is highest (Figures 1B and 8C).

It is tempting to speculate again whether *R. prolixus* NOS genes and their regulation by promoters may be capable of responding to both stimuli: one related to infectious processes and one responsible for regulating the synthesis of anti-hemostatic components in saliva. NOS expression in hemocytes was demonstrated to be suppressed after infection with *T. rangeli* [13], [14]. These data are consistent with the results obtained in the present study. The demonstration that ecdysone enhances the immune system activity of *R. prolixus* [40] strengthens the argument that this hormone may be responsible for modulating the expression of NOS in this insect. If hemocytes are responsive to ecdysone, it is possible that *T. rangeli* manipulates the generation of this hormone or some signaling pathway induced by it. Thus, a pre-adaptation of this parasite to inhibit the immune system of its host could have acted in a similar way, leading to regulation of the expression of anti-hemostatic molecules in saliva.

One of the difficulties in studies of cell signaling involving the infection of *T. rangeli* is the fact that this intracellular parasite uses various routes in order to colonize the invertebrate host and its several different compartments [41], [42]. Since no protocol has been developed for separating the parasites from the host cells, tissue extracts from infected *R. prolixus* were sometimes contaminated with cellular material from *T. rangeli*. Due to such technical constraints the proper analysis of control and infected groups was very difficult to be conducted. Thus, the use of molecules purified from the parasites should provide more reliable tests. Since many studies have shown the importance of the surface glycoconjugates of trypanosomatid parasites on the immune system of their hosts [21], [24], we tested the effects of such molecules on the expression of NOS in the salivary glands of *R. prolixus*. We observed that the administration of just 100 ng of Tr GIPL led to a decrease in NADPH-diaphorase activity of salivary glands extracts (Figure 2). This effect was not observed after administration of the same amount of GIPLs from *P. serpens* [27] or Mucins from *T. cruzi* [28] (Tc Mucins on Figure 2C). These results indicated that GIPLs were effectively recognized by the insect vector, generating a specific response to the infecting pathogen. Similar results were obtained by administration of *T. gondii* GIPLs and *P. falciparum* GIPLs [34], [46]. Tr GIPL induced a decrease in NADPH-diaphorase activity and also led to a decrease in NOS protein levels. The probe DAF-FM diacetate indicated that this decrease was sufficient to significantly reduce the generation of NO (Figures 4 and 5). Therefore, *T. rangeli* glycolipids reproduced the effects of infection with this organism on NOS. Previous studies suggested that infection with *T. rangeli* modulates the expression of NOS [13], [14]. However, the studies by Whitten et al., 2001 and Whitten et al. 2007 [13], [14] indicated that these parasites inhibit NO generation, but with major differences over the length of inhibition. This poses a novel challenge on the study of the mechanisms of NOS inhibition in invertebrate hosts, once it suggests that different mechanisms may operate over time during a single infection. If indeed *T. rangeli* is capable of modulating the expression of NOS in the salivary glands of *R. prolixus*, an understanding of the mechanisms used in this process may shed light onto the physiological regulation of this enzyme. For these reasons we tested the effects of infection with *T. rangeli* on the generation of NO in the salivary glands of *R. prolixus*. NADPH-diaphorase activity, Western-blots, immunolocalization experiments and NO imaging all demonstrated that *T. rangeli* reduces the level of NOS in the salivary glands of *R. prolixus*. In the studies by Garcia and colleagues (1994) [12] and Whitten et al (2007) [14], the generation of NO itself was not measured. Thus, the combination of NOS antibody and a fluorescent probe for NO in the present study provides the first direct measurement of NOS expression and NO generation in a model for infection by a trypanosomatid. Usually the opposite occurs, with few descriptions in the literature of models where NOS activity of hosts is inhibited [44], [45].

Our experiments showed that *T. rangeli* GIPLs reproduced the effects of infection with this parasite in terms of the increased total protein phosphorylation (Figure 8A). Surprisingly, GIPLs derived from *T. cruzi* epimastigotes induced similar effects on protein phosphorylation. In addition to reproducing the effects on protein phosphorylation, *T. cruzi* GIPLs also led to falling levels of NOS. Injection of *T. cruzi* into the hemolymph of these insects leads to an activation of their immune system and the elimination of these parasites [46]. As demonstrated by our group, the saliva of this insect enhances the infection of vertebrate hosts with *T. cruzi* [47]. Perhaps, *T. cruzi* glycolipids during the gut infection could eventually modulate the expression of some molecules in the saliva of *R. prolixus*, facilitating transmission of the parasite. Such point should be also addressed in the future. A description of the *Rhodniu*s sialome followed by microarray analysis of gene expression in trypanosomatid-infected insects may shed light onto this question in the future [48].

Studies in the literature have pointed out the role of protein tyrosine phosphatases (PTPs) in the regulation of NO synthesis [29], [30], [32]. The PTPs are key mediators of phosphotyrosine levels inside cells and are commonly manipulated by invading pathogens [49]. The involvement of other phosphatase classes, such as Ser/Thr phosphatases in *Rhodnius* NO synthesis was not evaluated in the present study. The demonstration of a protein phosphatase activity in the nucleus, especially in the heterochromatin of the epithelial cells of the salivary glands of *R. prolixus* led to the suggestion that these enzymes might regulate the expression of genes in these organs [50]. In addition, infection with *T. rangeli* inhibited the activity of an ecto-phosphatase present on the outer surface of *Rhodniuś* salivary glands [51]. Together, these results suggest that *T. rangeli* may induce changes in gene expression in the salivary glands of *R. prolixus* through mechanisms that involve the manipulation of phosphatase activities. For this reason, it is important to investigate the effects of glycolipids on phosphatase activity in salivary glands of this model. Tr GIPL led to a decrease in phosphatase activity of salivary gland extracts. Thus, both studies [50], [51] pointed out that *T. rangeli* infection relies on suppression of vector protein phosphatases. The silencing of bug PTPs by the aid of RNAi technology whenever *Rhodnius* genome is available may eventually provide information regarding vector refractoriness to protozoan infections. In conclusion, we have demonstrated for the first time that glycolipids are able to negatively modulate NOS expression, enhancing parasite transmission to the vertebrate host.

## Materials and Methods

### Ethics Statement

All animal care and experimental protocols were conducted following the guidelines of the institutional care and use committee (Committee for Evaluation of Animal Use for Research from the Federal University of Rio de Janeiro, CAUAP-UFRJ) and the NIH Guide for the Care and Use of Laboratory Animals (ISBN 0-309-05377-3). The protocols were approved by CAUAP-UFRJ under registries #IBQM001 and #IBQM011. All human participants involved in this study gave their verbal consent once blood was collected through a non-harmful procedure. The Committee for Ethics in Human Research at Hospital Universitário Clementino Fraga Filho specifically approved the experiments involving human participants (CEP-HUCFF/FM 213/07). Technicians dedicated to the animal facility at the Institute of Medical Biochemistry (UFRJ) carried out all aspects related to rabbit husbandry under strict guidelines to insure careful and consistent handling of the animals.

### Reagents

Ethylenediamine tetraacetic acid (EDTA), ethylene glycol tetraacetic acid (EGTA), flavin -adenine dinucleotide (FAD), culture medium RPMI-1640, LIT, NADPH, Tris, glycine, acrylamide, bis-acrylamide, Tetramethylethylenediamine (TEMED), Dimethyl sulfoxide (DMSO), Dithiothreitol (DTT), bovine serum albumin, sodium SO, okadaic acid, Folin reagent and pNPP were obtained from Sigma-Aldrich Company (St. Louis, MO, USA). The embedding medium Optimal cutting temperature compound (OCT) was obtained from Sabura Finetechnical Co. (Tokyo, Japan). (3-(4,5-Dimethylthiazol-2-yl)-2,5-diphenyltetrazolium bromide (MTT) was obtained from Calbiochem (La Jolla, CA, USA). Prestained Full-Range Rainbow molecular mass standards were obtained from Amersham Biosciences (Buckinghamshire, England). Ethanol, triton X-100 were obtained from Merck (Darmstadt, Germany). Anti-rabbit secondary antibody linked to alkaline phosphatase was obtained from Santa Cruz Biotechnology (Santa Cruz, CA, USA). Universal anti-NOS antibody was obtained from Oncogene Research Products Company (La Jolla, CA, USA). The Western blotting developer Western Blue was obtained from Promega Corporation (Madison, WI, USA).

### Insects

The experiments were conducted with nymphs of fourth and fifth stages of *Rhodnius prolixus* obtained from a colony at the Instituto de Bioquímica Médica at UFRJ, Brazil. These insects are kept at 28°C and 70% relative humidity and are fed with rabbit blood at regular intervals of three weeks.

### Parasite Culture and Insect Infection

Epimastigotes of the short form of the strain Macias were maintained in LIT medium-FCS (bovine liver infused 5 g/L, NaCl 4.4 g/L, KCl 0.4 g/L, glucose 2.2 g/L, Tryptose 5 g/L, NaH_2_PO_4_ 56 g/L, Yeast extract 5.0 g/L, 0.02 g Hemin/L and 10% fetal calf serum inactivated by heat) at 28°C. The cells were sub-cultured every three days according to Folly et al. (2003) [42]. Under these conditions the cells were in logarithmic growth phase and retained the ability to infect *R. prolixus.* Three days after the last replating the culture medium was centrifuged in a clinical centrifuge (International Equipment Company HN-SII, USA) for 15 min at 2500 rpm. The supernatant was discarded and cells resuspended in PBS-sucrose (0.1 M NaH_2_PO_4_ pH 7.4, 0.15 M NaCl and 1% sucrose). This procedure was repeated once and the parasites quantified in a Neubauer chamber. Two microliters of PBS-sucrose containing 5×10^4^ parasites/µL were injected into the ventral portion of the third segment of the thorax of fifth-stage nymphs of *R. prolixus* three weeks after its last blood meal. The control group was injected with the same volume of PBS-sucrose. All injections were performed using a Hamilton syringe with a capacity of 10 µL (H801 series). Typically, the syringe is kept immobilized on an iron stick and the insects were punctured on the edge of the syringe. On different days after infection, salivary glands were dissected from the nymphs and processed according to specific protocols for each experiment. The same procedure was used for the injection of isolated GIPLs. Tipically 50 pmoles GIPLs were injected with a microliter of GIPLs solutions at a concentration of 0.1 mg/µL. The GIPLs were diluted with deionized water and sonicated for 12 minutes in a bath sonicator brand Thorton once before the injections. Control groups were injected only with deionized water. Further conditions as described in [43].

### Glycoinositolphospholipids

*T*. *cruzi* GIPL used in the reported experiments was isolated from Tulahuen strain [26]; *P*. *serpens* GIPL [27]; and *T*. *cruzi* eGPI-mucin [28] were obtained as previously described. To purify *T*. *rangeli* GIPL, epimastigotes were grown in LIT (liver infusion tripticase) medium, supplemented with 20% of fetal bovine serum at 28°C with shaking (80 rpm) for 5 days. This was used to inoculate three liter flasks, containing 1 liter of the same medium under the same growth conditions. The cells were harvested by centrifugation, washed three times with 0.9% NaCl and frozen at −20°C. These procedures were repeated to get enough parasite cells to purify GIPLs. Briefly, frozen cells were thawed and extracted three times with cold water. The residue, remaining after the last centrifugation, was extracted with 45% (v/v) aqueous phenol at 75°C [53]. The aqueous layer was dialyzed, freeze-dried, dissolved in water, and applied to a column (2×100 cm) of Bio-Gel P-100. The excluded material was lyophilized and the dry residue shaken several times with chloroform/methanol/water (10∶10:3) for extraction of GIPL. The extracts were evaporated to dryness, under reduced pressure; the residue was dissolved in water and precipitated overnight at −20°C by addition of 5 volumes of methanol.

### Carbohydrate Analysis of Tr GIPLs

The monosaccharide composition of Tr GIPLs was determined according to Sweeley et al. [54]. After methanolysis with 0.5 M HCl in methanol (18 h, 80°C), the mixture was extracted with hexane and the methanolic phase neutralized with Ag2CO3. The products were N-acetylated with acetic anhydride, dried, and treated with bis-(trimethylsilyl)trifluoroacetamide (BSTFA)/pyridine (1∶1, v/v, 1 h, room temperature).

Trimethylsilyl derivatives were analyzed by gas chromatography (GC) using a DB-5 fused silica capillary column (25 m×0.25 mm i.d.) with hydrogen (10 psi) as the carrier gas. The column temperature was programmed from 120°C to 240°C at 2°C/min.

### Analysis of Inositol and Glucosamine of Tr GIPLs

The Tr GIPLs were treated with 3 M HCl in methanol for 18 h at 80°C. The methanolysates were dried under a stream of N_2_, the resulting residue was dissolved in 1.0 ml of aqueous 6 M HCl and heated for 18 h at 105°C. After hexane extraction, to remove fatty acids, the aqueous layer was lyophilized. The products were N-acetylated with acetic anhydride, dried, and treated with bis-(trimethylsilyl)trifluoroacetamide (BSTFA)/pyridine (1∶1, v/v, 1 h, room temperature). Trimethylsilyl derivatives were analyzed by gas chromatography (GC) using a DB-5 fused silica capillary column (25 m×0.25 mm i.d.) with hydrogen (10 psi) as the carrier gas. The column temperature was programmed from 120°C to 240°C at 2°C/min.

### Lipid Analysis of Tr GIPLs

After methanolysis of the Tr GIPL with methanolic-HCl (18 h at 80°C), fatty acid methyl esters (FAMEs) were extracted with hexane. The extracts were concentrated and analyzed by GC after derivatization with BSTFA/pyridine as described in carbohydrate analysis. The column temperature was programmed from 180°C to 280°C at 3°C/min. Peaks were identified by their retention time compared with authentic standards and by gas chromatography coupled mass spectrometry (GC-MS). For the analysis of long-chain bases, GIPCs were methanolyzed (1 M methanol-HCl made 10 M with respect to water) [55]) for 18 h at 80°C. After adjusting the pH to about 11 with aqueous NaOH, long-chain bases were extracted with diethyl ether. The extracts were washed with water, dried with anhydrous sodium sulfate, evaporated to dryness, dissolved in methanol, and N-acetylated with acetic anhydride (18 h, room temperature in the dark). The product was treated with BSTFA/pyridine and analyzed by GC and GC-MS as described for the FAMEs.

### Dissection and Homogenization of Salivary Glands

Nymphs of *R. prolixus* previously anesthetized in ice had their salivary glands dissected in TBS buffer (10 mM Tris-HCl pH 8.0, 150 mM NaCl). The homogenization of salivary glands was performed using a 2 µL buffer for each pair of glands. These samples were frozen in liquid N_2_ three times and sonicated twice, in cycles of 12 min in a Thornton sonicator bath. Subsequently, samples were centrifuged for 5 min at 11000 *g*. The precipitated material was discarded and the supernatant referred to as salivary gland extract. The protein content of these samples was measured using a colorimetric method [56]. Extracts routinely contained 15 pairs of salivary glands. This material was designed to test several enzyme activities such as NADPH-diaphorase, phosphotyrosine phosphatase, and also for Western blots. Using this method both the major salivary glands and the accessory gland were homogenized together. In order to simplify the reading of the protocols below, the term “salivary gland” includes the main and accessory glands.

### NADPH-diaphorase Activity of Salivary-gland Extracts

Salivary-gland homogenates were assayed for NADPH-diaphorase activity according to Ribeiro and Nussenzveig (1993) [4]. Briefly, the extracts were adjusted to a final concentration of 0.5 pairs/µL in TBS buffer. The tests were performed in buffer containing 10 mM Tris-HCl pH 8.0, 0.05 M NaCl, Triton X-100 0.1%, 1 mM CaCl_2_, 5 mM FAD, NADPH 1 mM and MTT at 0.5 mg/mL. The MTT reduction was monitored colorimetrically at 540 nm for 30 min at 37°C. Readings were taken on a Thermo Max ELISA reader (Molecular Devices) using Thermo Max software for the measurement of enzyme kinetics. Each test was performed in triplicate, using a pair of salivary glands (150 µg protein) per experimental point.

### Western Blotting

Salivary-gland extracts (100 µg protein) were diluted in sample buffer (2% SDS, 5% β-mercaptoethanol, 62.5 mM Tris-HCl pH 6.8, 1% glycerol and 12.5 mg/mL bromophenol blue). After boiling for 5 min, samples and prestained Full-Range Rainbow molecular mass standards (250, 160, 105, 75, 50, 35, 30, 25, 15, 10 kDa) were used in a 7.5% bis-acrylamide gel (15×15×0.1 cm) with 0.1% SDS. Protein extracts were fractionated using a constant current of 20 mA [57], then electrotransferred from the gel to a nitrocellulose membrane at 200 mA for 120 min at 4°C in Tris-HCl 25 mM pH 8.3, 192 mM glycine and 20% methanol (v/v) [58]. The membrane was blocked with 10 mM Tris-HCl pH 8.0 containing 0.15 M NaCl, 0.05% Tween-20 and 2% bovine serum albumin fraction V (TBSTA) for 18 h at 4°C. The next day, the blocking buffer was discarded and the membrane incubated with polyclonal anti-universal NOS (anti-NOS, Oncogene) diluted 1∶1000 in TBSTA for 1 h at room temperature. Then, the membrane was washed three times for 5 min with 15 mL of TBSTA and incubated for 30 min at room temperature with anti-rabbit IgG (conjugated with alkaline phosphatase) diluted 1∶10,000 in TBSTA. The membrane was washed three times for 5 min each with 15 mL of TBS. Finally, the antigen-antibody complex was revealed by adding 7 mL of the Western Blue developer (Pierce). After development, the reaction was stopped with two washes of 50 mL of distilled water.

### Immunolocalization of NOS

Twenty days after parasite infection or injection with PBS-sucrose, nymphs had their salivary glands dissected as described above. After dissection, glands were fixed whole in 50 mL of PBS-4% paraformaldehyde for 2 h. After fixation the material was washed three times with 500 mL of PBS for 2 min and then incubated in 500 mL of PBS-20% sucrose (0.1 M NaH_2_PO_4_ pH 7.4, 0.15 M NaCl and 20% sucrose) at 40°C for 24 h. Samples were taken from the buffer and placed in trays of 0.5 cm^2^, containing half the final volume of the embedding medium OCT. The glands were carefully positioned and covered with the rest of the embedding medium. This material was then frozen in liquid N_2_ for later use. The samples were processed in a cryostat at −180°C, generating slices 10 µm thick, which were placed in quadruplicate on glass slides. These slides had been previously soaked in alcohol, dried, soaked in 1% gelatin solution and dried again to allow the cryosectioning. Slides with cryosections were subsequently thawed and the OCT removed by incubating the same slides with TBS for 10 min. Then these sections were permeabilized with 0.1% Triton-TBS for 10 min. After these procedures, cryosections were blocked in TBSTA solution for 1 h at 4°C and immunolocalization was performed using anti-NOS AB-1 (Oncogene) diluted 1∶100 in TBSTA for 1 h. After one hour the samples were incubated in TBSTA for 30 min, then incubated with anti-rabbit secondary antibody conjugated with fluorescein for 2 h. Next 3 washes of 5 min each with TBS-T and three washes with TBS were conducted. After the last wash a drop of n-propylgalactate was added to each blade which was then closed with a coverslip and kept in the dark at 6°C until image acquisition. The images were acquired on an Axioplan epifluorescence microscope (Zeiss) at a magnification of 63x. Images were then treated equally in X and Adobe Photoshop 5.5 (Adobe). Controls were performed with uninfected glands or incubated in medium devoid of primary antibody. All incubations and washes mentioned above were performed in a final volume of 200 µL.

### NO Imaging

DAF-FM diacetate (Molecular Probes) is a readily permeable probe for NO which diffuses through the cytoplasmic membrane. Upon reaching the cytoplasm of cells, DAF-FM diacetate is deacetylated by esterases, becoming DAF-FM, which is slightly fluorescent. Once NO reacts with DAF-FM it is converted into a derivative that fluoresces around 160 times more than the original molecule. The wavelengths for excitation/emission maxima are 495/515 nm respectively. Salivary glands (freshly dissected and intact) were incubated with *Rhodnius* Ringer (130 mM NaCl, 8 mM KCl, 2 mM CaCl_2_, 8.6 mM MgCl_2_, 10 mM NaHCO_3_, 4 mM NaH_2_PO_4_, 30 mM glucose). The DAF-FM diacetate was added in the dark at a final concentration of 25 mM and the glands were incubated for one hour at 25°C and then washed with 1 mL of Ringer’s to remove excess DAF-FM diacetate. Finally, the material was taken to the epifluorescence microscope for visual inspection at the above-mentioned wavelengths. Each experiment was performed with 10 pairs of glands per experimental group.

### RNA Extraction and PCR Analysis

Eighteen pairs of salivary glands of *R. prolixus* (per experimental point) were homogenized in TRIzol ® (Invitrogen). Total RNA was isolated from these samples by following the manufacturer’s instructions. One microgram of RNA from each sample was digested with DNAse according to the manufacturer’s protocol (Fermentas) and cDNA was generated using MultiScribe™ Reverse Transcriptase kit (Applied Biosystems). Quantitative real time PCR was performed using Sybr Green PCR Master Mix (Applied Biosystems). *Rhodnius prolixus* primers sequences used were: NOS: Forward 5′ GCTTTCCATTCCCAGGTGTTAT 3′; Reverse 5′ TTGCCAAACGTTGAAGCTACA 3′; endogenous control used was ribosomal protein 18S: Forward 5′ TGTCGGTGTAACTGGCATGT 3′; Reverse 5′ TCGGCCAACAAAAGTACACA 3′ [60], [61]. Data were analyzed by comparative Ct Method (Applied Biosystems user bulletin #2). Also in some experiments we have conducted semi-quantitative PCR assays were also conducted as indicated in Results and Figure legends. In this case primers sequences used were: NOS: Forward 5′CCT TCA GTA GGC GTT CTT C 3′, Reverse 5′ TACGACGGCTACAGTCAAAT 3′; endogenous control used was *R. prolixus* ribosomal protein 18S : Forward 5′ GTT GGTATTGATGTACGCTGGA 3′, Reverse 5′ CCTACGGAAACCTTGTTACGA 3′.

### Anticoagulant Activity Measured by Activated Partial Thromboplastin Time (aPTT) Assay

The ability of *R. prolixus* salivary gland extracts to inhibit plasma coagulation was assessed by aPTT assay on an Amelung KC4A coagulometer (Labcon, Heppenheim, Germany). Human blood samples were collected from healthy donors in 3.8% trisodium citrate (9∶1, v/v), and platelet-poor plasma were obtained by further centrifugation at 2,000*×g* for 15 min. Plasma (50 µL) was incubated with 10 µL of salivary glands at various concentrations (suspension in PBS buffer) for 2 min at 37°C, followed by addition of the aPTT reagent (cephalin plus kaolin, 50 µl). After 1 min at 37°C, plasma clotting was initiated by the addition of 100 µL of 25 mM CaCl_2_ and the time for clot formation was then recorded. The anticoagulant activity of extracts of salivary gland was calculated as the fold increase in coagulation time of plasma incubated with 1 mg of salivary gland extract relative to control plasma [61].

### Endogenous Phosphorylation of Proteins of Salivary Gland Extracts

Salivary gland extracts were incubated in reaction medium for kinases (20 mM MgCl_2_, 150 mM NaCl, 50 mM Tris-HCl pH 8.0 and 100 µM ^32^P-ATP [1,000 cpm/pmol]) for an hour at 37°C. The reaction was stopped by adding sample buffer (2% SDS, 5% β-mercaptoethanol, 62.5 mM Tris-HCl pH 6.8, 1% glycerol and 12.5 mg/mL bromophenol blue). The samples were boiled for 5 min and then fractionated on a 10% Bis-acrylamide gel (15×15×0.1 cm) using a constant current of 20 mA in the presence of SDS [57]. After this procedure the gels were stained with a dye solution (Coomassie Blue G-250 1g/L, methanol 20% v/v, acetic acid 10% v/v) for one hour. Then the gels were bleached (methanol 40% v/v, acetic acid 10% v/v). The gels were coated with 5% glycerol solution (v/v) and dried in cellophane at room temperature, then covered with an X-ray film in the dark and stored at –80°C for 30 days. At the end of this time the films were developed, scanned and fixed. The gels were also scanned and their images were worked together, making use of Adobe Photoshop 5.5 (Adobe).

### Apyrase Activity of Salivary Gland Extracts

Apyrase activity of 0.1 mg of total salivary gland extract was determined by measuring the generation of Pi after addition of ATP (final concentration 2 mM) to the reaction medium (50 mM Tris-HCl pH 7.5, 0.15 M NaCl, 5 mM CaCl_2_). The medium was incubated for 15 min at room temperature and the reaction was stopped by the addition of buffer containing 1.25% ammonium molybdate, 2.5 N sulfuric acid, 90 mM Na_2_SO_3_, 110 mM Na_2_S_2_O_5_ and 1-amino-2-naphthol 4–8 mM. Pi generated was quantified by according to Fiske and Subbarow (1925) [62]. Readings were taken at 660 nm in a Thermo Max ELISA reader (Molecular Devices) using Thermo Max software and a standard curve of NaH_2_PO_4_. A curve with salivary-gland protein samples of 0.1, 0.2, 0.3, 0.4, 0.5 and 1.0 µg was performed to test whether there was depletion of substrate in 15 min. The reaction remained linear up to 0.5 µg of protein.

### Phosphotyrosine Phosphatase Activity

Homogenates with the equivalent of a pair of salivary glands of the fifth-stage nymphs were diluted in 100 mM Tris, 100 mM glycine, 100 mM citrate and 100 mM acetate pH 5.0. pNPP was added to a final concentration of 0.1 mM and the samples were incubated at 37°C for one hour in the presence or absence of 1 mM SO, a classic inhibitor of phosphotyrosine phosphatases. At the end of the reaction the medium was alkalinized with 2 N NaOH. The dephosphorylation reaction was measured colorimetrically at 405 nm on a Thermomax Elisa reader. The SO-sensitive activity was calculated as the fraction of total enzyme activity that was inhibited in the presence of 1 mM SO [63].

### Statistical Analysis

All experiments were performed in triplicate. The results are presented as the mean and standard error of the mean SE. Normalized data were analyzed by one-way analysis of variance (ANOVA) or Student t-test as indicated in Figure legends using the software GraphPad Prism.
